# Diel versus time-integrated (daily) photosynthesis and irradiance relationships of coral reef organisms and communities

**DOI:** 10.1371/journal.pone.0208607

**Published:** 2018-12-17

**Authors:** Yvonne Sawall, Eric J. Hochberg

**Affiliations:** Coral Reef Ecology and Optics Laboratory (CREOL), Bermuda Institute of Ocean Sciences (BIOS), St. George’s, Bermuda; University of Barcelona, SPAIN

## Abstract

The most important source of energy to tropical shallow water coral reefs is light, the transformation of which ultimately limits reef biomass and growth. Therefore, measurements of productivity (primary production, P) for benthic reef organisms and communities are critical to understand reef functioning. Short-term (minutes to hours) P measurements of reef photosynthesizers virtually always produce the classic hyperbolic tangent (or similar) P-E (irradiance) relationship, with P rapidly rising to a saturation point as E increases. Longer-term (days to weeks), larger-scale investigations of natural reef communities typically do not explore P-E relationships, but the few that do show no saturation of *time-integrated* P with high *time-integrated* E. In this paper we present a modeling study to reconcile this apparent contradiction. We used 52 published short-term (*instantaneous*) P-E curves of organisms (corals, algae) and communities (corals, mixed corals and algae) from different reefs in the Indo-Pacific and the Caribbean, each coupled with 928 diel light curves comprising a wide range of cloud cover scenarios. The diel light curves provided *instantaneous* E at 1-minute intervals, from which we calculated corresponding *instantaneous* P using the different published P-E relationships. We integrated both variables to calculate *time-integrated* E and P. *Time-integrated* E varied up to 18-fold due to changes in cloud cover and season. We found that, despite routine saturation of *instantaneous* P, day-scale P-E relationships were near linear in all cases, with slightly decreased linearity in cases where instantaneous light saturation occurred very early during the day. This indicates that the Functional Convergence Hypothesis (FCH) developed by terrestrial ecologists may also apply for reef photosynthesizers. The FCH states that despite short-term light saturation, plants on average do not absorb more light than they can use, since resource allocations are strictly coordinated and tailored towards an optimal use. Thus, there is no contradiction: At the growth time scale (≥ day), P should be expected to be a near linear function of E. One implication is that reef P can be estimated using rapid optical measurements, as opposed to traditional, laborious respirometry methods. The requirement going forward is to derive appropriate values for light-use efficiency, which is the rate at which the plant or community converts absorbed light into fixed carbon.

## Introduction

Light is the most important source of energy for shallow water benthic coral reef communities, where corals and algae are highly productive (e.g., [1, 2]). Most corals (hard and soft) strongly depend on the productivity of their photosynthesizing endosymbionts, dinoflagellates of the genus *Symbiodinium* (zooxanthellae), which cover most of the energy demands of corals and hence determine coral growth (calcification in the case of hard corals; [3–6]). Free-living algae form another important group of primary producers in reefs and include, for example, crustose coralline red algae (CCA), “fleshy” calcifying (e.g., *Halimeda*) and non-calcifying algae and turf algae. CCA contribute to reef growth as well as to substrate stabilization (e.g., by cementing coral rubble; [7]), while fleshy macroalgae and turf algae serve as an important food source for herbivores [8, 9]. Microalgae (e.g., diatoms) and cyanobacteria contribute to reef productivity, as well [10, 11]. Since primary production (P) of shallow water reef organisms ultimately determines reef biomass and growth [12, 13], measurement of P for individuals and communities is of high priority to understand reef functioning.

Current approaches to measure P of coral reef organisms and communities in-situ are all based on respirometry. These include organism [14, 15] or community enclosures [16, 17], flow respirometry over reef communities [18,19], and the gradient flux approach [20, 21]. These methods each feature different logistical restrictions and are generally very laborious, which makes large-scale (>1 km) and repetitive measurements (e.g., for monitoring purposes) very difficult. Another challenge is interpreting in-situ P measurements under light conditions that vary day to day; the very nature of photosynthesis means that changes in sky conditions lead to changes in absolute rates of P. This makes it difficult to conduct comparative studies, where measurements are made for reefs in different locales or across seasons/years. Optical tools, as employed to measure P for terrestrial plant and ocean phytoplankton ecosystems via remote sensing (e.g., [22–25]), do not have these limitations and may therefore be a promising alternative to measure coral reef P [26]. An important prerequisite to expand optical-based P measurement to coral reefs is that reef photosynthesizers follow the same principle of photosynthesis adjustment as plants in other biomes, which would be reflected in a (near-) linear relationship of *time-integrated*, day-scale P and irradiance (E) of reef organisms and communities.

P measurements for terrestrial systems via remote sensing are based on the concept of light-use efficiency (LUE) introduced by Monteith [27] and Monteith and Moss [28], where LUE is the ratio of P to absorbed photosynthetically active radiation (APAR). Consequently, P can be calculated by multiplying measured APAR and *known* LUE (e.g., [26]):

$$P=LUE\int_{\lambda =400}^{700}E_{d}(\lambda )A(\lambda )d\lambda =LUE\times APAR,$$ (1)

where spectral downwelling plane irradiance [*E*_d_(*λ*)] is the light flux (photons area^−1^ time^−1^ wavelength^−1^) incident to the organism or community at a given wavelength (*λ*), and spectral absorptance [*A*(*λ*)] describes the fractional amount of light absorbed by the community (non-dimensional). LUE has units oxygen or carbon photon^−1^. The optical terms are integrated across the wavelengths of photosynthetically active radiation (PAR; 400–700 nm). *A*(*λ*) is determined using reflectance spectroscopy, where a spectrometer measures the fraction of light reflected by the organism or community; the fraction *not* reflected is absorbed [*A*(*λ*) = 1 –*R*(*λ*)]. Similar expressions have also been developed with respect to marine systems [29, 30]. (Table 1 lists parameters used in this publication and provides a brief description for each.)

**Table 1:** Description of parameters used or mentioned in the publication.

Parameter	Description
P	Primary production
P_max_	Maximum primary production
NP	Net primary production
GP	Gross primary production
E	Irradiance
E_k_	Saturation irradiance, derived from P-E curves
E_max_	Maximum irradiance
LUE	Light-use-efficiency; P / APAR [e.g., O_2_ photon^−1^]
APAR	Absorbed photosynthetically active radiation
*E*_d_(*λ*)	Spectral downwelling plane irradiance (photons area^−1^ time^−1^ wavelength^−1^)
*A*(*λ*)	Spectral absorptance, the fraction of incident light absorbed by the organism(s) or substrate
*R*(*λ*)	Spectral reflectance, the fraction of incident light reflected by the organism(s) or substrate
*λ*	Wavelength

The linear form of Eq (1) is in apparent contradiction to the very well known non-linear, hyperbolic tangent (and similar) relationship between P and E [31]:

$$P=P_{max}tanh(E/E_{k}),$$ (2)

where E is (photosynthetically available) irradiance, E_k_ is the saturation irradiance, and P_max_ is the horizontal asymptote of photosynthetic capacity. Under Eq (2), P increases rapidly as E increases to E_k_; beyond E_k_, P increases slowly until reaching P_max_.

The contradiction between Eqs (1) and (2) is explained by the time frame of reference. Eq (2) considers *instantaneous* P and E at seconds to hours, while Eq (1) considers *time-integrated* P and E (or APAR) at the day scale and longer. These time-scale-varying patterns in the P-E relationship are borne out by observation in terrestrial plant systems [32, 33]. The reason for the different P-E relationships is that it is not energetically cost-effective for plants to adjust pigment levels at time scales of milliseconds to an hour, where light intensity can rapidly vary by more than an order of magnitude. It is advantageous, however, for plants to adjust pigment levels to best utilize the long-term, prevailing light field. A classic example is the difference between light- and shade-acclimated corals [34]. The result is that, although light saturation may occur on short time scales and on small spatial scales, plants *on average* do not absorb more light than they can use. That is, plants optimize their photosynthetic capacity to prevailing conditions, e.g., general availability of light, nutrients, CO_2_, and temperature [33].

This ability of plants to adjust photosynthetic capacity forms the basis of the Functional Convergence Hypothesis (FCH), which states that resource allocations are strictly coordinated and tailored towards an optimal use [33, 35] and that these fundamental optimization processes are similar across different species [36, 37]. If the FCH holds true, for a given set of environmental (temperature, nutrients, etc.) conditions, LUE—the plant’s capacity to convert absorbed light to fixed carbon—is expected to be relatively constant over time, independent of normal variations in day-to-day light availability. LUE should be also similar for different species that share similar life histories and resource limitations. Ultimately, a constant LUE in Eq (1) means that P is mainly determined by APAR, which varies with light availability and the plant’s capacity to harvest light (e.g., abundance of light-harvesting pigments).

The aim of this study is to examine whether coral reef benthic organisms and communities feature a (near-) linear relationship of *time-integrated* P and E (or APAR). To date, there are no suitable APAR—and hence LUE—data available, but we can assume that APAR is proportional to E. Thus, P-E curves should show the same type of relationship as P-APAR curves. This assumption is based on the fact that pigment concentrations typically change slowly over time and, hence, the organism’s ability to absorb light remains practically constant over the course of a day and between successive days.

In order to obtain *time-integrated* P and E (or APAR) curves, we used the following modeling approach. First, we identified published, empirical *instantaneous* P-E curves for a range of different coral reef benthic organisms and communities. Next, we acquired several years’ worth of solar irradiance (E or PAR) diel curves at 1-minute intervals. Then, we applied *instantanteous* PAR to each P-E curve, thus modeling *instantaneous* P. We integrated these data to calculate daily P and E. The resulting *time-integrated* P thus encompassed a wide range of E scenarios (overcast to sunny, and short winter days to long summer days). We found that the *time-integrated* P-E relationships (daily rates), in contrast to *instantaneous* P-E relationships, were near linear in all cases.

## Material and methods

Fifty-two published *instantaneous* P-E curves were chosen, representing a number of (although not all) studies conducted on a wide range of benthic reef organisms (corals and algae) and communities (coral dominated, turf algae dominated, sand with microalgae), as well as environmental conditions. The latter was determined by season, depth (0 to 65 m), distance from land, biogeographic region (Red Sea, Great Barrier Reef, Central Pacific, Caribbean, Mediterranean) and latitude (14° to 44°). Studies were either conducted under natural light conditions over a daily cycle or under artificial light conditions in the laboratory using different light intensities. The publications and relevant information for all *instantaneous* P-E curves are listed in S1 Table, which includes species/community type, location, depth, date of measurement, environmental conditions, P_max_, E_k_ and maximum E (E_max_).

**Fig 1:**
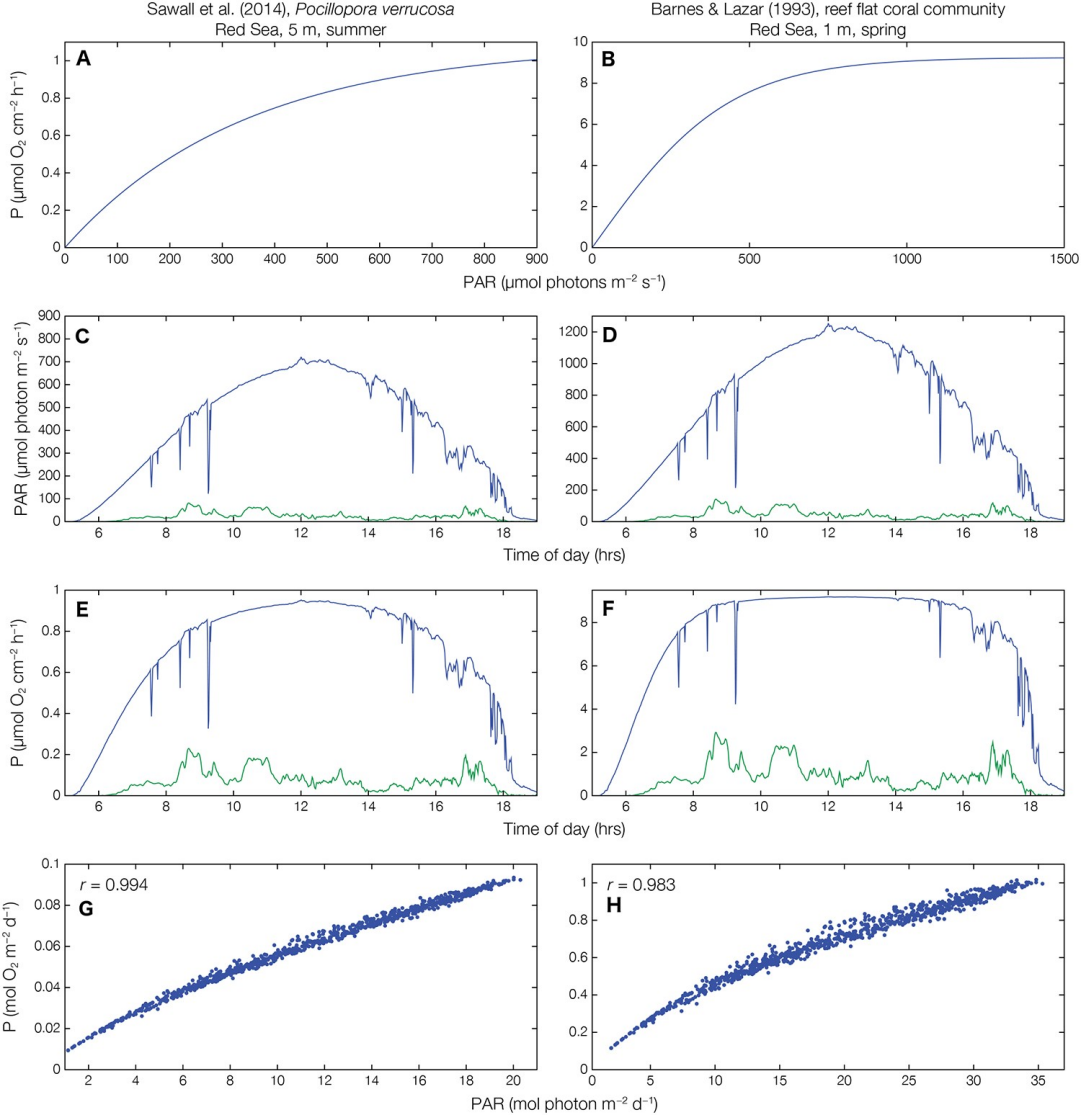
Results of the different steps of the model for a late saturating [15] and early saturating [2] *instantaneous* P-E curve. *Instantaneous* P-E curves reconstructed from the equations and parameters described in the respective publications (see specifics in Table 1) (**A, B**). Maximum (green line) and minimum (green line) *instantaneous* PAR of the Bermuda light curves adjusted to local maximum PAR of published P-E curve (**C, D**). Derived *instantaneous* P corresponding to adjusted PAR at different times of day and under different cloud cover scenarios (**E, F**). *Time-integrated* P-E curves representing daily rates of P and daily PAR for 928 different days with different cloud cover scenarios (**G, H**).

To gain a large range of *time-integrated* (daily) E values representing different cloud intensities and day lengths, we gathered all the available, valid diel insolation curves during 2012–2014 (total 928 curves) from the National Weather Service pyranometer stationed at L. F. Wade International Airport in Bermuda (S2 Table). These curves included all conditions from virtually clear to heavy overcast throughout all seasons and covered an 18-fold range of daily E. The wide range of daily E in Bermuda likely exceeds the range occurring in other coral reef regions, since Bermuda is situated at a particularly high latitude (32.3°N) for warm water coral reefs. However, use of this range allows a conservative modeling approach, providing day-scale light variability beyond that expected in most locations of this study, except the Mediterranean. For each P-E simulation, we scaled the maximum E value across all diel curves to 110% of the reported (or estimated) E_max_ (μmol photons m^−2^ s^−1^). The resulting diel E curves had the correct magnitudes for each given study, and cloud effects were based on actual cloud patterns (examples in Fig 1C and 1D).

To determine P, we simply applied the modeled E to the appropriate measured *instantaneous* P-E curve (examples in Fig 1A and 1B). This produced 928 diel curves of *instantaneous* P (examples in Fig 1E and 1F). The diel E and P curves were numerically integrated (trapezoid rule), providing 928 rates at the day scale for each study. Plotted against each other, this resulted in conservative *time-integrated* P-E curves (examples in Fig 1G and 1H), with daily E for each case ranging from values slightly higher than measured in-situ (110% of E_max_) to values somewhat lower than expected in the corresponding season (6% of E_max_). Expected minimum values are ~10–15% of maximum daily E, based on Falter et al. [38], who found a 7-fold change (14%) in daily E within one week due to changes in cloud cover.

## Results

### Model demonstration

The results of the different steps of the *time-integrated* P-E modeling are demonstrated in Fig 1 with two examples, one for an *instantaneous* P-E curve saturating late (left column) and one saturating early (right column) in the day. Both examples show the modeled variability of *instantaneous* E and P, with E ranging between 100% on a cloudless day to 6% on a heavily overcast day. The *time-integrated* P-E curves show a near-linear relationship in both examples; only the lower ends of the curves (low light intensities) exhibit slightly steeper slopes, in particular where the *instantaneous* P-E curves saturate earlier (Fig 1; see [2]). The earlier saturating *instantaneous* P-E curves also result in a slightly higher scatter of points in the *time-integrated* P-E curve.

### Model results

**Fig 2:**
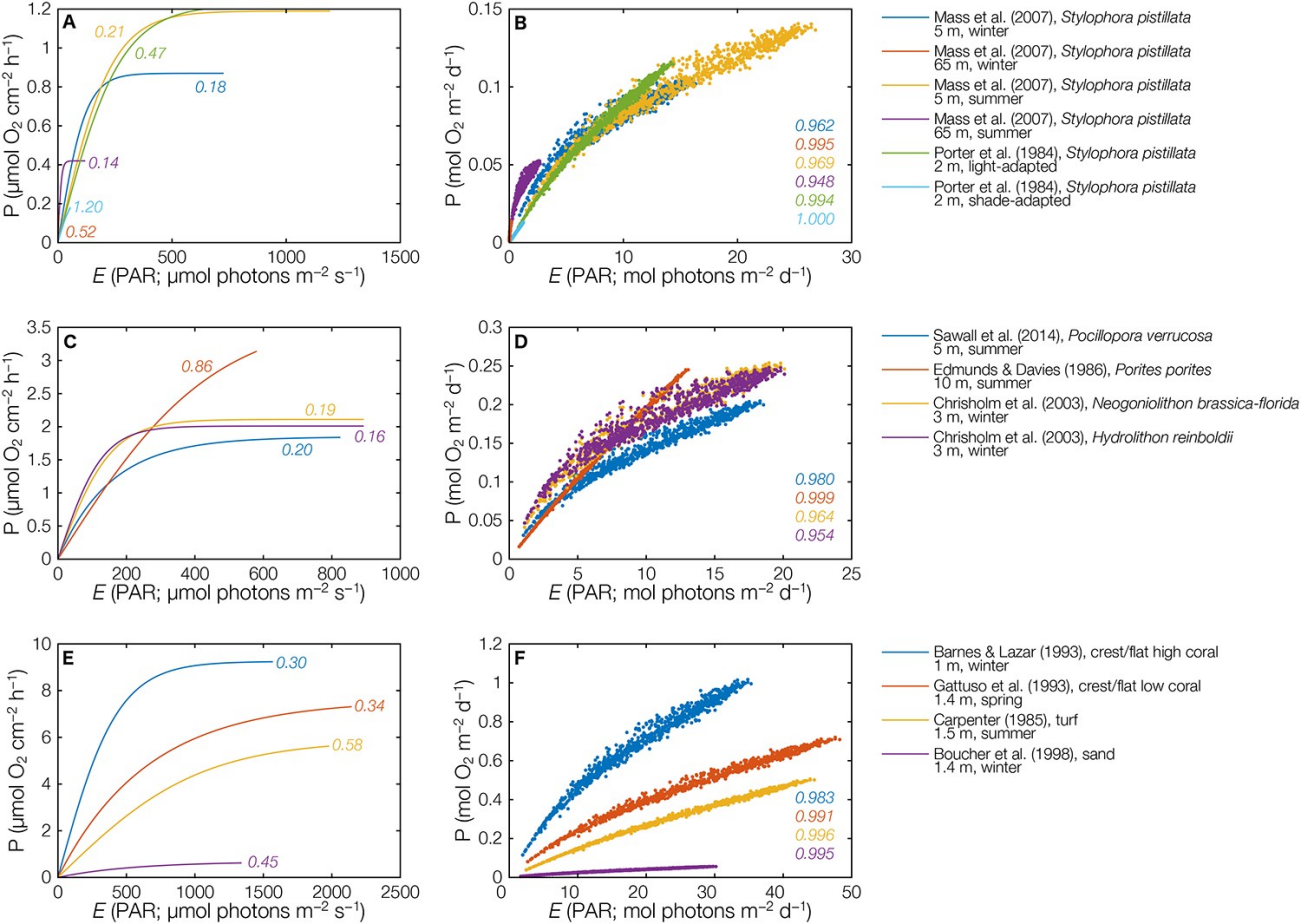
*Instantaneous* P-E curves (left column: A, C, E) and corresponding *time-integrated* P-E curves (right column: B, D, F) of the coral species *Stylophora pistillata* (A, B), of different individual reef corals and algae (C, D) and of different reef communities (E, F). P = photosynthesis or productivity, E = irradiance, PAR = photosynthetically available radiation, values within the graphs A, C and E = E_k_/E_max_, values within the graphs B, D and F = correlation coefficient *r*.

**Fig 3:**
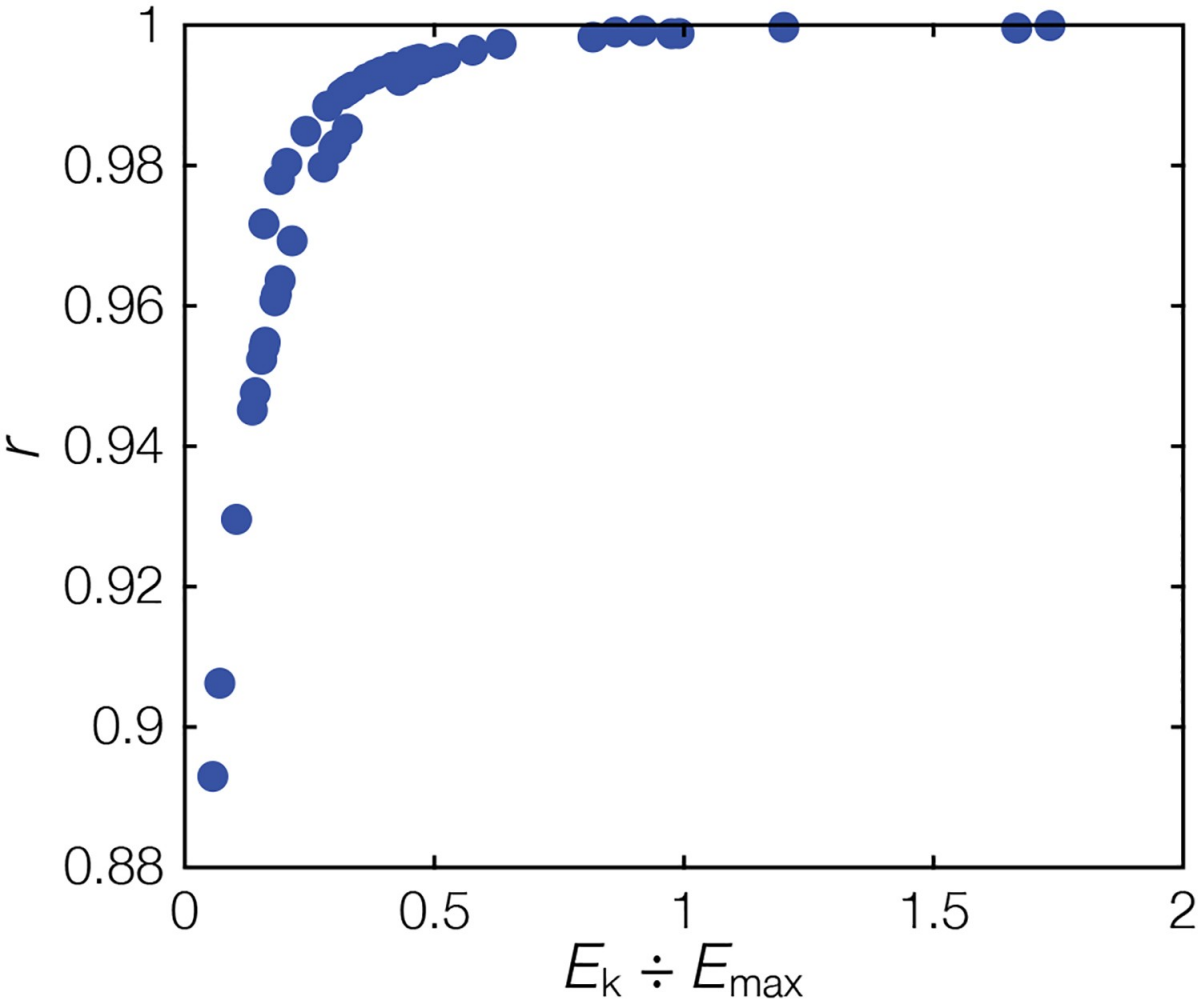
Relationship of light saturation / maximum irradiance ratio (E_k_/E_max_) of the 52 published *instantaneous* P-E curves and the correlation coefficient (*r*) of the corresponding *time-integrated* P-E curves.

All *time-integrated* P-E curves revealed statistically significant linear relationships with *r* > 0.89 (p < 0.001, S1 Table). Those *time-integrated* P-E curves with *r* < 0.98 (comparatively lower linearity) exhibited a slightly steeper initial slope, where daily E was ≤25% of maximum daily PAR (Fig 2B, 2D and 2F). This is related to the rather low E_k_ of the corresponding *instantaneous* P-E curve (Fig 2A, 2C and 2E), as indicated by the relationship between the *instantaneous* E_k_/E_max_ ratios and *r* of the *time-integrated* P-E slopes (Fig 3). In particular, when E_k_/E_max_ dropped below 0.2, which occurs in 13 of the 52 curves, the linearity of the *time-integrated* P-E curve decreased markedly (Fig 3).

### Comparison of P-E curves of different species and communities in different habitats

In order to visualize the *time-integrated* P-E relationship between different species, communities, and environmental settings, we plotted a selection of P-E curves. Fig 2A shows the *instantaneous* P-E relationships for the coral *Stylophora pistillata* in the Gulf of Aqaba (Red Sea) in two different seasons, in different depths, and under different local light conditions due to shading (light exposed versus shaded). Fig 2B shows the corresponding *time-integrated* (daily-scale) P-E relationships, which are linear in all instances when E_k_ is higher than ~25% of E_max_, such as at 65 m depth in winter (E_k_/E_max_ = 0.52) and in shaded location at 2 m depth (E_k_/E_max_ = 1.2).

Fig 2C and 2D present *instantaneous* and *time-integrated* P-E curves of different species, including the corals *Pocillopora verrucosa* (Red Sea, 5 m, summer) and *Porites porites* (Jamaica, 10 m, summer) and the crustose coralline red algae (CCA) *Neogoniolithon brassica-florida* and *Hydrolithon reinboldii* (both Great Barrier Reef, 3 m, winter). Here, the differences between species with low and high E_k_/E_max_ values are particularly evident, where the two CCA species saturate very early (E_k_/E_max_ ~0.18), resulting in a large scatter of points and a steeper initial slope. In contrast, *P*. *porites* at 10 m depth saturates late (E_k_/E_max_ ~0.86), resulting in a strong linear P-E relationship throughout.

Fig 2E and 2F show the *instantaneous* and *time-integrated* P-E curves of different shallow water whole reef communities (1–1.5 m depth). Those include (*i*) a reef flat close to the reef edge in the Gulf of Aqaba, Red Sea, with a high coral cover, (*ii*) a reef flat with a comparatively low coral cover and increased algal abundance in Moorea (transect from the reef edge [31% live coral cover] into the lagoon [<2%]), (*iii*) a turf algae community in the US Virgin Islands, and (*iv*) a microalgae community inhabiting a sandy area in the Moorea lagoon. The coral-dominated community exhibits the steepest slope and the largest scatter, owing to a comparatively lower E_k_/E_max_ value. The two macroalgae communities show very similar slopes, both slightly less than the coral community, and the low-biomass microalgae community has a very shallow P-E slope.

## Discussion

**Fig 4:**
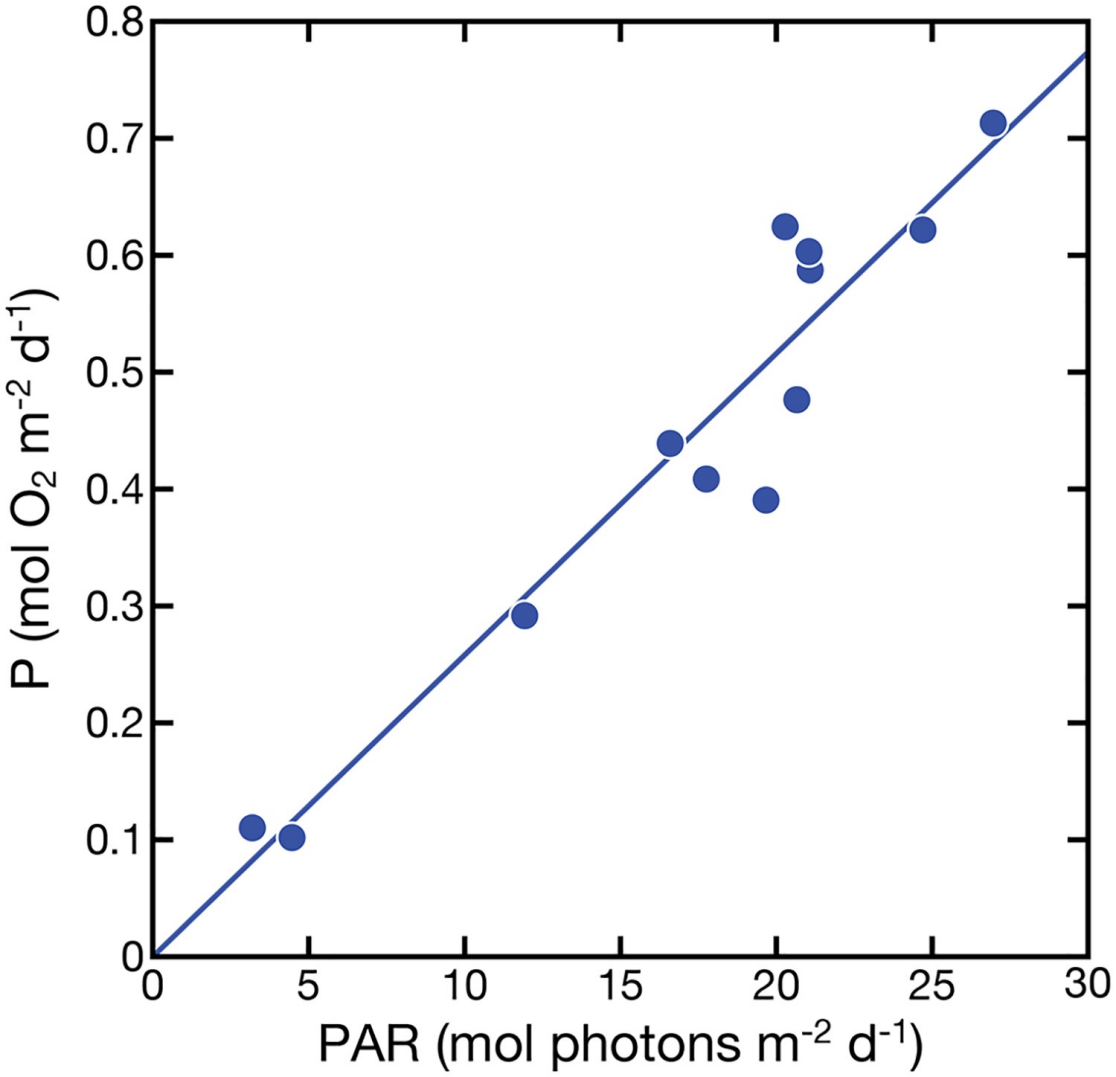
Measured daily gross photosynthesis rate P vs. PAR for the Kaneohe Bay reef flat showing that P does not saturate at higher irradiances at the day scale. R^2^ = 0.92. Data for P and PAR provided by courtesy by J. Falter. Data is presented in different way in Falter et al. 2011 [38].

Without exception, all modeled *time-integrated* P-E curves revealed a much stronger linear relationship than their respective *instantaneous* P-E curves within their natural range of light intensities. Day-scale P-E relationships for organisms/communities with a low E_k_ value relative to E_max_ (early saturation) showed a slightly steeper initial slope, which transitioned into a less steep slope that was strongly linear at daily E > ~25% of maximum. Conversely, organisms/communities with higher E_k_ relative to E_max_ exhibited a stronger linear fit of *time-integrated* P-E curves across the entire range of daily E. This was equally valid for individual colonies of corals and coral fragments, for different algal species, and for different coral reef benthic communities measured in-situ as well as under laboratory conditions. Hence, the pattern is independent of individual, community, and environment. Our modeled results are corroborated by a study of Falter et al. [38], whose data exhibit a strong linear relationship between measured daily gross production rates and measured daily E on an algal-dominated reef flat in Kaneohe Bay, Hawaii, where daily E varied 7-fold (Fig 4, r = 0.96).

These results indicate that reef organisms (corals and algae) most likely optimize photosynthesis to the same degree as terrestrial plants—at the day scale and longer—and therefore possess an important prerequisite of functional convergence. This optimization allows short-term light saturations at high mid-day irradiance, where an excess of absorbed light is dissipated through photo-protective mechanisms (as heat or fluorescence). However, *on average*, the photosynthetic machinery is tuned so that no more light is absorbed than the plant requires or is able to process efficiently. Theoretically, this would result in equal daily LUEs independent of light availability [32, 33, 35]. In other words, reef organisms and communities scale their investment in photosynthetic capacity to average light availability [35]. The fact that this can be observed across various reef species points toward the second part of the FCH, which implies that LUEs are similar across different species [33, 36, 37].

Optimization of light absorption and utilization (CO_2_ fixation) is, on one hand, determined by the carbon requirement of the plant or coral, which includes respiration to maintain cell functioning, production of biomass (growth), reproduction, and defense against predation and pathogens. On the other hand, it is limited by resource availability such as nitrogen, CO_2_ and light [39–43]. For example, in many systems, nitrogen, an element essential for various enzymes and structural components, is a limiting resource [44, 45]. Assuming optimal nitrogen utilization, an organism should allocate as much nitrogen to the photosynthetic apparatus (e.g., light harvesting pigments, RuBisCo, enzyme D1, etc.) as necessary to meet the demand of carbon acquisition to maintain plant functioning under prevailing nitrogen and light conditions [46]. As another example, light limitation leads to stronger nutrient allocation to the photosynthetic machinery, in particular for the construction of light harvesting pigments and pigment-protein complexes, in order to maintain photosynthetic gain [34, 47, 48, 49]. In turn, excess light availability can lead to resource allocation for mechanisms that reduce light absorption (e.g., production of fluorescent pigments in corals; [50]) and in the decomposition of light harvesting pigments and complexes [34, 47]. These examples demonstrate that the adjustments of the photosynthetic apparatus, and hence P, are the product of the optimized use of resources. P can therefore also be regarded as an ecological integrator, meaning that photosynthetic performance integrates all prevailing growth conditions [33, 35].

Interestingly, the near linearity of the *time-integrated* P-E relationship seems to be even more pronounced in a reef system than in a terrestrial system. Haxeltine and Prentice [32] showed that in terrestrial systems the linearity is particularly evident when P is measured on a larger spatial scale, meaning on the level of a canopy instead of on the leaf level (part of a plant). In contrast, we have found a near-linear *time-integrated* P-E relationship for coral fragments (e.g., [15, 51]) and even for small coral nubbins [52]. A potential explanation for this is based on differences in the complexity of a tree and a coral or alga. A tree is divided into different compartments (e.g., roots, stem, branches, leaves adapted to shaded and to light-exposed conditions), where each compartment has a certain function, and only the tree as a whole can work efficiently adjusted to local (resource) conditions. Reef photosynthesizers, on the other hand, are structured on a much smaller scale. Coral colonies consist of a number of small, more or less independent entities, the polyps, which are often only few millimeters in diameter. Hence, a fragment or even a small nubbin of coral consists of a number of polyps harboring millions of photosynthesizing zooxanthellae and is therefore able to work as efficiently (per unit area) as a whole coral colony or even a coral community.

It should be mentioned that, while most P-E curves used for this study represent net productivity (NP), some represent gross productivity (GP) (S1 Table). In Fig 2, this includes *P*. *porites* in Jamaica (Fig 2C and 2D; [3]), the coral-dominated reef flat in the Gulf of Aqaba (Fig 2E and 2F; [2]), and the microalgal community of the sand in a lagoon of Moorea (Fig 2E and 2F; [10]). However, employing either NP or GP has no influence on the shape and slope of the P-E relationship, only on the position of the modeled data points, since the published GP rates were usually calculated by simply adding night respiration rates to the NP rates. (Note, that NP versus GP would have an effect on calculated LUE and the hypothesized similarities between LUEs between species; [35]).

The result of our model is promising with respect to optics-based (APAR-based) P measurements in coral reefs, although some potential limitations have been identified for early-light-saturated reef organisms and communities. In cases where light saturation occurs later in the day (E_k_/E_max_ > 0.25), the near-linearity of the *time-integrated* P-E relationship implies a steady and reliable LUE across all light intensities. Early-light-saturated organisms and communities (E_k_/E_max_ < 0.25), in contrast, exhibit a steeper slope between day-scale P and E at low light intensities. A single LUE would be less representative across the full range of day-scale E, though still useful, especially for day-scale E ≥ ~25% of full range. Early-light-saturated organisms and communities also exhibit more scatter in the *time-integrated* P-E relationship, which will inevitably result in less accurate APAR-based P rates. The error estimated from our modeled *time-integrated* P-E curves can be as high as 30% in extreme cases (e.g., shallow-water crustose red algae [7]).

Keeping these limitations in mind, optics-based P measurements may have a range of advantages over traditional respirometry-based techniques. Namely, optical measurements are independent of local topography, and they can be used across spatial scales. The next step toward optics-based P measurements is to determine actual LUEs for different reef photosynthesizers. These LUEs should be based on GP instead of NP in order to gain LUEs independent of respiration. LUEs based on NP would otherwise underestimate the actual LUE and would reflect potential variability in respiration. Furthermore, it is known that P rates and photo-inhibitory processes vary between morning and afternoon at equal E (e.g., [53, 54, 55]). Hence, daily LUE should not be derived from *instantaneous* P-E curves, but by integrating multiple *instantaneous* P rates measured throughout the entire day. If LUE is fairly constant between species as suggested by the FCH [35], variability in P would thus derive from concomitant variability in APAR, which is dependent on an organism’s capacity to absorb light [*A*(*λ*)]. Dubinsky et al. [40] found that about 33% of E is absorbed by the Red Sea coral *Stylophora pistillata* under natural low-nutrient conditions, while absorption increased up to 85% under nutrient enrichment. Enriquez et al. [56] found absorption rates up to more than 90% in healthy *Porites branneri* in the Caribbean. Hochberg et al. [57] have shown that, worldwide, typical coral and algal *R*(*λ*) is ~10%, which equates to *A*(*λ*) of ~90%. LUE, in contrast, is defined by the ability of the organism to process the captured light, meaning the conversion of energy gained by photons into cellular energy (ATP) and carbon fixation via the electron transport chain and within the Calvin Cycle. This is a rather standardized process across species, although variability may occur. For example, temperature or water velocity changes may influence enzyme activity and CO_2_ availability, respectively [58]. These changes may affect different species to different degrees, and those species may differ in their abilities to adjust to these changes. However, if a rather narrow range of LUEs can be proven and/or LUE can be modeled based on environmental conditions, optics-based P measurements, even on remote sensing scale, are very feasible. This would have the potential to advance our understanding on reef functioning over space and time significantly.

## Supporting information

S1 Table Summary of information of all *instantaneous* P-E curves used for modeling and results of corresponding *time-integrated* P-E curves. Gross photosynthesis (GP) and net photosynthesis (NP) refers to what was measured in the corresponding study. Maximum photosynthetic rate (P_max_), light saturation point (E_k_), maximum irradiance reaching measured individuals or communities (E_max_). Slope of *time-integrated* P-E curve is the ratio of daily P / daily E in mol O_2_ m^-2^ d^-1^ / mol photons m^-2^ d^-1^ in cases where *instantaneous* P was reported as μmol O_2_ cm^-2^ h^-1^. In case *instantaneous* P was reported in another unit, the original unit was maintained. Note that the slope is not the actual light-use efficiency (LUE), since LUE describes the ratio of *time-integrated* P / APAR, not P / PAR.
(XLSX)

S2 Table Mat lab file containing all the normalized diel PAR curves used for modeling. The variable "Normalized_PAR" contains 928 diel curves of normalized PAR. Each column is a single diel curve. These curves represent the range from cloudy days with short photoperiod to sunny days with long photoperiod. These values can be scaled by E_max_ for use in each specific instantaneous P-E curve, as given in S1 Table. The variable "Time_of_Day" is a single column vector with time of day from midnight to midnight in decimal days, at one-minute intervals. The file was written using Matlab version R2017a.
(MAT)
